# Seed Mucilage Improves Seedling Emergence of a Sand Desert Shrub

**DOI:** 10.1371/journal.pone.0034597

**Published:** 2012-04-12

**Authors:** Xuejun Yang, Carol C. Baskin, Jerry M. Baskin, Guangzheng Liu, Zhenying Huang

**Affiliations:** 1 State Key Laboratory of Vegetation and Environmental Change, Institute of Botany, Chinese Academy of Sciences, Beijing, China; 2 Jiangxi Academy of Forestry, Nanchang, China; 3 Department of Biology, University of Kentucky, Lexington, Kentucky, United States of America; 4 Department of Plant and Soil Sciences, University of Kentucky, Lexington, Kentucky, United States of America; University of Tartu, Estonia

## Abstract

The success of seedling establishment of desert plants is determined by seedling emergence response to an unpredictable precipitation regime. Sand burial is a crucial and frequent environmental stress that impacts seedling establishment on sand dunes. However, little is known about the ecological role of seed mucilage in seedling emergence in arid sandy environments. We hypothesized that seed mucilage enhances seedling emergence in a low precipitation regime and under conditions of sand burial. In a greenhouse experiment, two types of *Artemisia sphaerocephala* achenes (intact and demucilaged) were exposed to different combinations of burial depth (0, 5, 10, 20, 40 and 60 mm) and irrigation regimes (low, medium and high, which simulated the precipitation amount and frequency in May, June and July in the natural habitat, respectively). Seedling emergence increased with increasing irrigation. It was highest at 5 mm sand burial depth and ceased at burial depths greater than 20 mm in all irrigation regimes. Mucilage significantly enhanced seedling emergence at 0, 5 and 10 mm burial depths in low irrigation, at 0 and 5 mm burial depths in medium irrigation and at 0 and 10 mm burial depths in high irrigation. Seed mucilage also reduced seedling mortality at the shallow sand burial depths. Moreover, mucilage significantly affected seedling emergence time and quiescence and dormancy percentages. Our findings suggest that seed mucilage plays an ecologically important role in successful seedling establishment of *A. sphaerocephala* by improving seedling emergence and reducing seedling mortality in stressful habitats of the sandy desert environment.

## Introduction

Seeds have special adaptations to different environments, and thus germination and subsequent seedling emergence may be altered by a wide range of environmental factors such as water, temperature, light, allelochemicals, mechanical interference and microbial pathogens [1], [2]. Survival of new plants in arid environments is mainly determined by mechanisms that ensure germination and seedling development at the right time and in a suitable place [3]. Seedling establishment is the critical stage controlling plant survival in these environments [1], [4]. In arid and semiarid areas, sand dunes are characterized by spatio-temporal variation in water availability [5], and the temporal changes in water availability have a profound impact on plant survival [6], [7]. Thus, the success of seedling establishment of desert plants depends on seedling emergence response to an unpredictable precipitation regime.

In sand dune habitats, sand burial is a crucial and frequent environmental stress factor, and the microenvironment varies with sand burial depth. For example, sand near the surface is moistened even by a light rainfall, but evaporation is high. In contrast, sand in deep layers is moistened only after a relatively heavy rainfall, but the moisture can persist longer. After a precipitation event, seeds at different burial depths are exposed to different moisture conditions. Therefore, seed germination and seedling emergence are determined by the depth of seed burial in the sand [8]. The plants inhabiting sand dunes have developed special adaptations to the frequently occurring sand burial, which has strong selective pressures on fitness through natural selection [9], [10]. Sand burial is reported to decrease seedling emergence [10]–[12] and enforce seed dormancy [10], [13]–[15] in a number of species. However, it also is proposed to confer some advantages for seeds. For example, sand burial can prevent seed predation by surface foragers such as ants and beetles [16], [17], provide more suitable microsites for germination and increase seedling survivorship because of instant access to moisture and insulation of the root system from high temperatures and desiccation [9]. Therefore, investigating the response of seedling emergence to sand burial is important for understanding the adaptation of plants to sand dune systems.

The extremely diverse external surface of seed coats of angiosperms reflects a multiple adaptation to seed dispersal and germination in different environments [18]. Upon imbibition of water, seeds of many species produce a pectinaceous mucilage (myxospermy) that has multiple functions in seed maturation [19], [20], dispersal [21], [22] and germination [19], [23]–[25]. However, it remains unknown how mucilage affects seedling establishment in the sand dune environment, in which precipitation and sand burial are two selective factors for the survival and distribution of dune plants in arid and semi-arid areas [4].

*Artemisia sphaerocephala* Kraschen. (Asteraceae) is one of the most important pioneer plants of the moving and semi-stable sand dunes in the deserts and steppes of northwest and north-central China [26]. This shrub has strong resistance to drought, cold and saline-alkaline soil conditions and is widely used for vegetation restoration in this area because it protects the sand from erosion by wind. During seed maturation, a polysaccharide is secreted through the epidermal cells of the fruit wall and accumulates in layers of mucilage. Involucral bracts of inflorescences protect achenes from wetting by rain until after dispersal [26], [27]. Our recent studies have shown that seed mucilage of this species is multifunctional. It can help maintain DNA integrity with the aid of desert dew or with small amounts of rain in the growing season [27], [28]. Therefore, seed mucilage can maintain a functional soil seed bank [28] and aid seed germination in osmotically stressful and saline habitats [25]. Biodegradation of seed mucilage promotes early seedling growth in barren sand dunes, in association with a large soil microbial community that supplies substances promoting seedling establishment [29]. Nevertheless, little is known about the role of mucilage in seedling emergence, especially in relation to sand burial.

*A. sphaerocephala* experiences both desiccation and sand burial during seedling emergence in its natural habitat of moving sand dunes. We hypothesized that the mucilage on the outer surface of *A. sphaerocephala* achenes can enhance seedling emergence in the unpredictable desert precipitation regime and under conditions of sand burial. Thus, we asked the following questions: (1) Can mucilage enhance seedling emergence of *A. sphaerocephala* in different precipitation regimes? and (2) Can mucilage enhance seedling emergence under sand burial? Answers to these questions will advance our understanding of the ecological functions of the seed mucilage in the life history strategies of plants in the natural environment.

## Results

### Soil moisture

**Figure 1:**
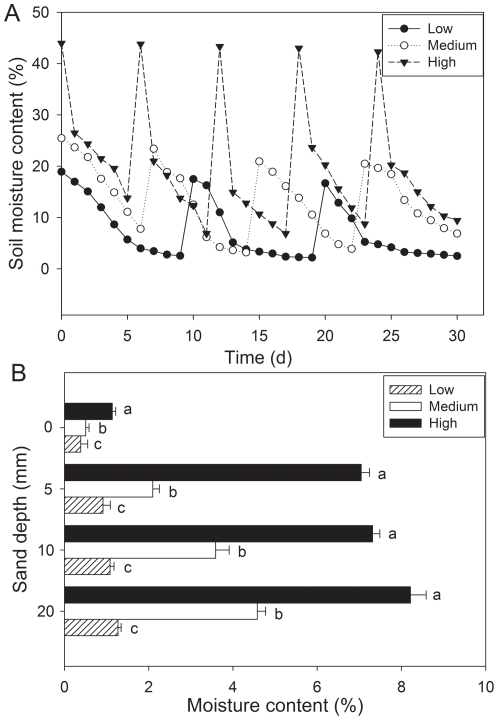
Soil moisture dynamics (A) and moisture content at different sand depths at end of seedling emergence experiment (B) in three irrigation regimes. Different lowercase letters indicate significant difference between irrigation regimes at a sand burial depth. Horizontal bars in B represent ±1 SE. n = 4.

Irrigation regime (*F*_2,24_ = 449.99, *P*<0.001), sand depth (*F*_3,24_ = 216.35, *P*<0.001) and their interactions (*F*_6,24_ = 25.41, *P*<0.001) significantly affected sand moisture content. Sand moisture was higher at the high irrigation regime than at the intermediate and low regimes. In high precipitation regime, sand moisture in pots ranged from 6.9% to 44.0%. Minimal sand moisture in irrigation regimes of medium precipitation and low precipitation was 3.2% and 2.2%, respectively. Maximal sand moisture in medium and low precipitation was 25.5% and 18.9%, respectively (Figure 1A). Sand moisture content at different sand depths where achenes were sown were also higher in high precipitation than that of medium and low precipitation and moisture content increased with increasing sand depths (Figure 1B).

### Seedling emergence

**Figure 2:**
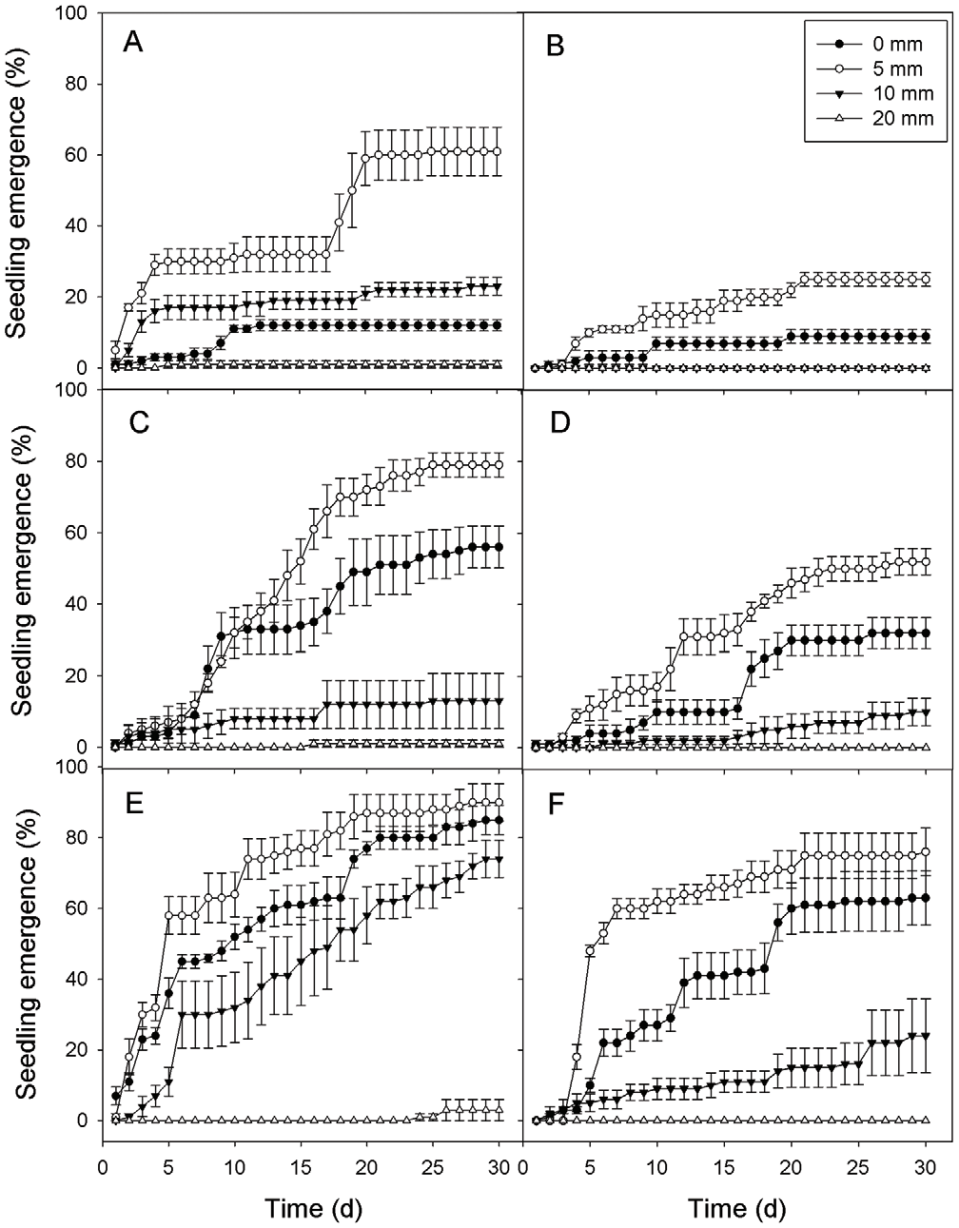
Cumulative seedling emergence of two types of *A. sphaerocephala* achenes at different sand depths in three irrigation regimes. A, intact achenes in low irrigation; B, demucilaged achenes in low irrigation; C, intact achenes in medium irrigation; D, demucilaged achenes in medium irrigation; E, intact achenes in high irrigation; F, demucilaged achenes in high irrigation. Vertical bars represent ±1 SE. n = 4.

For both types of *A. sphaerocephala* achenes, cumulative seedling emergence was lower in low precipitation than in medium and in high precipitation during the 30-d experiment (Figure 2). Regardless of irrigation regime and mucilage manipulation, 5 mm sand burial depth had the highest seedling emergence.

Three-way ANOVA showed that final seedling emergence was significantly affected by irrigation regime, mucilage manipulation, sand burial depth and their interactions except the interaction between mucilage manipulation and irrigation regime (Table 1). In low precipitation, seedling emergence of intact achenes at 0, 5 and 10 mm burial depths was significantly higher than that of demucilaged achenes at the same depths (*P*<0.05; Figure 2A, 2B). Seedling emergence percentages of intact achenes were significantly higher than those of demucilaged achenes at 0 and 5 mm burial depths in medium precipitation (*P*<0.05; Figure 2C, 2D). In high precipitation, seedling emergence of intact achenes was significant higher than that of demucilaged achenes at 0 and 10 mm burial depths (*P*<0.05), but it was not significant at other burial depths (Figure 2E, 2F).

**Table 1:** Three-way ANOVA of effects of mucilage manipulation, irrigation regime and sand burial depth on seedling emergence and mortality, T_50_ and on seed quiescence and dormancy of *A. sphaerocephala*.

	Final emergence				Mortality				T_50_				Quiescence percentage				Dormancy percentage			
Source	Type III SS	df	*F*-value	*P*-value	Type III SS	df	*F*-value	*P*-value	Type III SS	df	*F*-value	*P*-value	Type III SS	df	*F*-value	*P*-value	Type III SS	df	*F*-value	*P*-value
Irrigation regime (IR)	3.04	2	94.01	**<0.001**	9.25	2	84.46	**<0.001**	6.42	2	5.84	**<0.01**	3.52	2	112.08	**<0.001**	0.06	2	1.73	0.185
Mucilage manipulation (MM)	1.27	1	78.54	**<0.001**	3.16	1	57.62	**<0.001**	4.75	1	8.65	**<0.01**	0.18	1	11.38	**<0.01**	0.47	1	28.94	**<0.001**
Burial depth (BD)	11.08	3	228.91	**<0.001**	1.75	2	15.95	**<0.001**	4.45	3	2.70	0.055	4.56	3	96.84	**<0.001**	0.64	3	13.14	**<0.001**
IR×MM	0.08	2	2.31	0.107	0.06	2	0.56	0.577	0.35	2	0.32	0.728	0.31	2	9.88	**<0.001**	0.36	2	11.18	**<0.001**
IR×BD	1.22	6	12.63	**<0.001**	0.96	3	5.87	**<0.01**	7.45	6	2.26	0.052	0.64	6	6.76	**<0.001**	1.27	6	13.08	**<0.001**
MM×BD	0.32	3	6.58	**<0.01**	1.80	2	16.41	**<0.001**	2.13	2	1.94	0.155	0.65	3	13.80	**<0.001**	0.28	3	5.68	**<0.01**
IR×MM×BD	0.35	6	3.60	**<0.01**	0.06	3	0.37	0.779	1.33	3	0.81	0.495	0.58	6	6.15	**<0.001**	0.60	6	6.22	**<0.001**
Error	1.16	72			2.63	48			28.00	51			1.13	72			1.16	72		

### Seedling mortality

**Figure 3:**
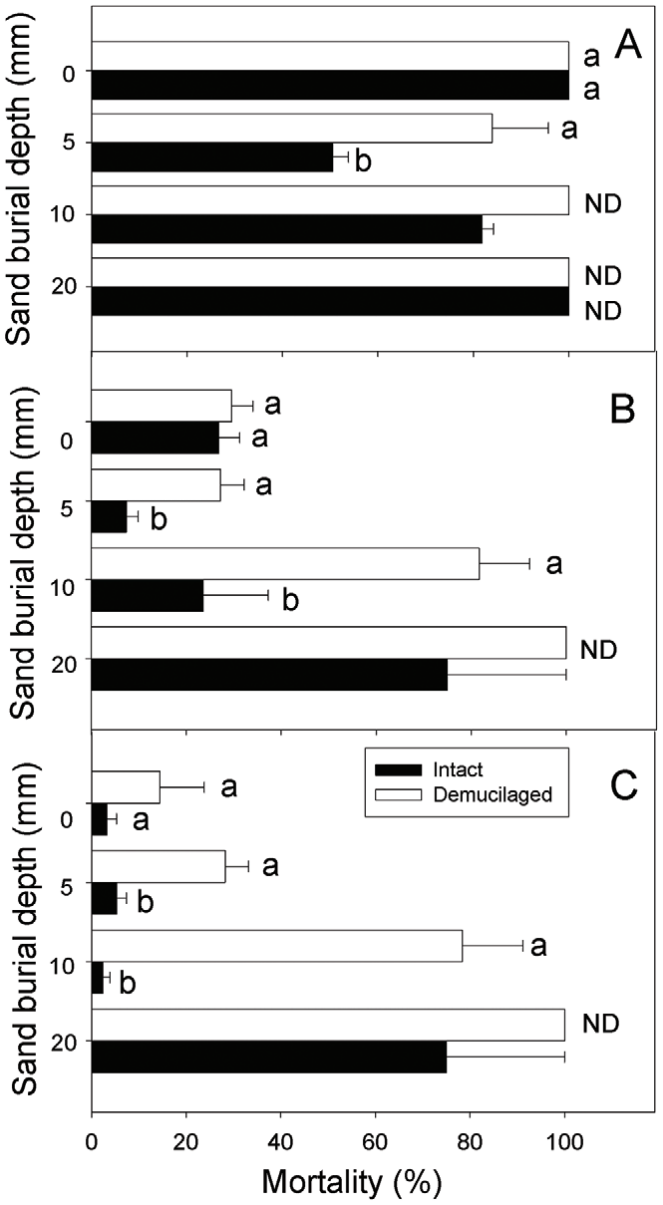
Seedling mortality of two types of *A. sphaerocephala* achenes at different sand burial depths in the irrigation regimes of low (A), medium (B) and high (C). Different lowercase letters indicate significant difference between two mucilage manipulations at a sand burial depth. ND, not detected. Horizontal bars represent +1 SE. n = 4.

Main factors of mucilage manipulation, irrigation regime and sand burial depth significantly affected seedling mortality, and the interactions between irrigation regime and burial depth and between mucilage manipulation and sand burial depth were also significant (Table 1). In low precipitation, seedling mortality of intact achenes at 5 mm sand burial depth was significantly lower than that of demucilaged achenes (Figure 3A). Seedling mortality between the two mucilage manipulations was significantly different at 5 and 10 mm burial depths in medium precipitation (Figure 3B). In high precipitation, seedling mortality between the two mucilage manipulations differed significantly at 0 to 10 mm burial depths (Figure 3C).

### Rate of seedling emergence

**Figure 4:**
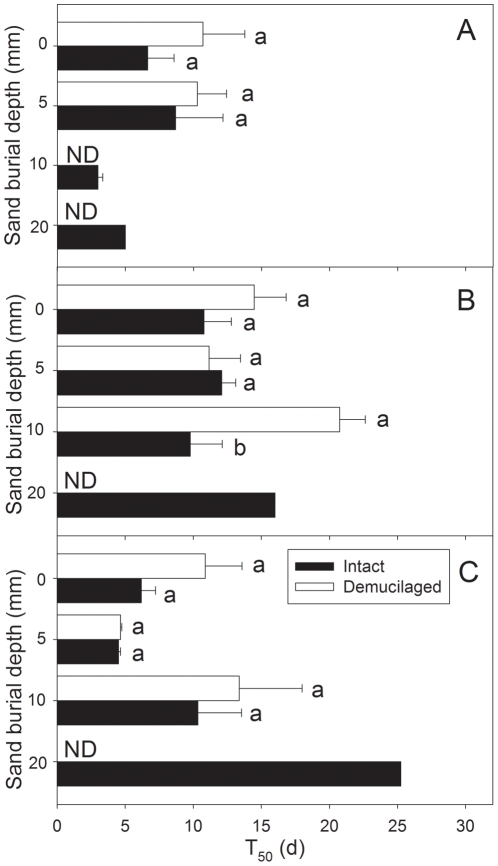
T_50_ of two types of *A. sphaerocephala* achenes at different sand burial depths in the irrigation regimes of low (A), medium (B) and high (C). Different lowercase letters indicate significant difference between two mucilage manipulations at a sand burial depth. Horizontal bars represent +1 SE. n = 4.

Three-way ANOVA showed that time to reach 50% emergence (T_50_) was significantly affected by irrigation regime and mucilage manipulation (Table 1). Generally, T_50_ of intact achenes was lower than that of demucilaged achenes. T_50_ in low and high precipitation regimes was lower than that in medium precipitation (Figure 4A, 4B, 4C). In addition, the effects of burial depth and interactions between irrigation regime and burial depth were marginally insignificant (Table 1). In low precipitation, T_50_ at deeper burial depths (10 and 20 mm) was lower than that at shallower burial depths (0 and 5 mm), but the opposite was the case in high precipitation. In medium precipitation, T_50_ was similar at all burial depths (Figure 4A, 4B, 4C).

### Quiescent achenes

**Figure 5:**
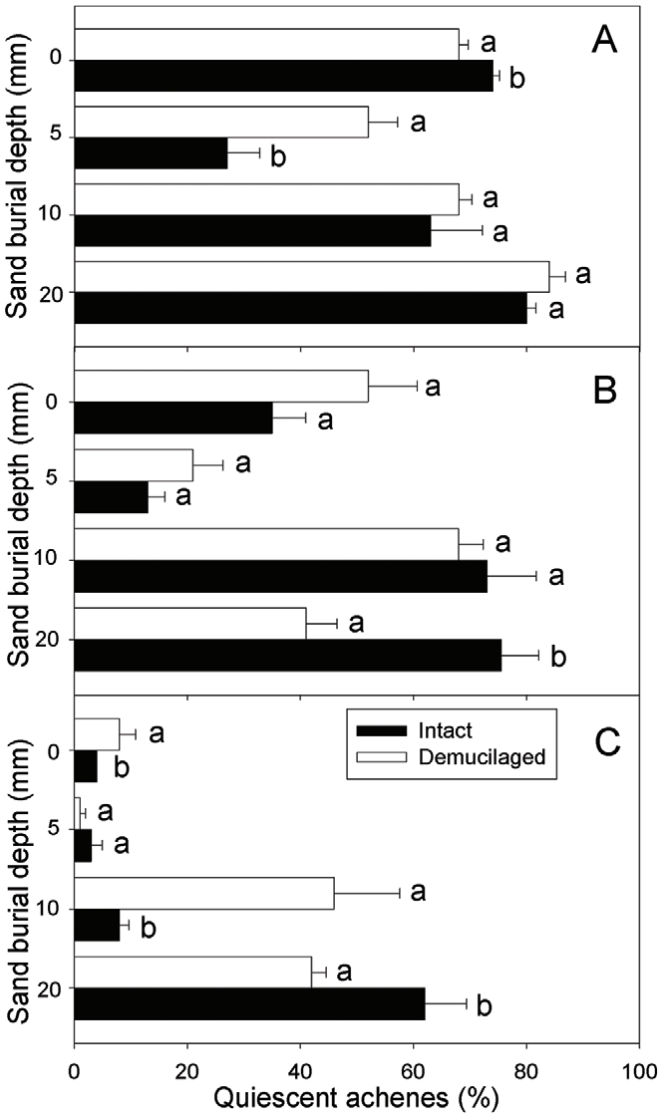
Quiescence percentage of two types of *A. sphaerocephala* achenes at different sand burial depths in the irrigation regimes of low (A), medium (B) and high (C). Different lowercase letters indicate significant difference between two mucilage manipulations at a sand burial depth. Horizontal bars represent +1 SE. n = 4.

Irrigation regime, mucilage manipulation, sand burial depth and their interactions significantly affected percentage of quiescent seeds (Table 1). Quiescence percentage of intact achenes at 0 mm burial depth was higher than that of demucilaged ones in low precipitation, but the opposite is the case at 5 mm (Figure 5A). In medium precipitation, quiescence percentage of intact achenes was significantly higher than that of demucilaged achenes at 20 mm burial depth, but this difference was not significant at the other burial depths (Figure 5B). Quiescence percentage was more variable between the two mucilage manipulations in high precipitation, being significantly lower for intact achenes at 0 and 10 mm burial depths. However, quiescence percentage of intact achenes at 20 mm burial depth was significantly higher than it was at 0 and 10 mm burial depths, and there was no difference between the two mucilage manipulations at the 5 mm burial depth (Figure 5C).

### Dormant achenes

**Figure 6:**
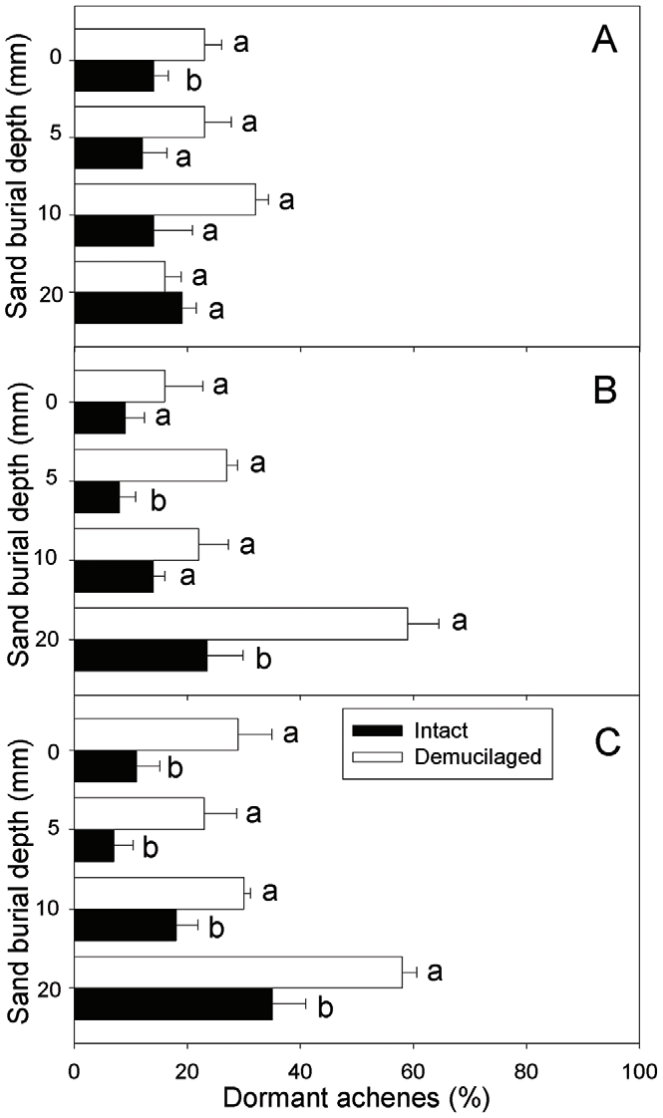
Dormancy percentage of two types of *A. sphaerocephala* achenes at different sand burial depths in the irrigation regimes of low (A), medium (B) and high (C). Different lowercase letters indicate significant difference between two mucilage manipulations at a sand burial depth. Vertical bars represent +1 SE. n = 4.

Three-way ANOVA revealed that dormancy percentage was significantly affected by mucilage manipulation, sand burial depth and all interactions of the three factors (Table 1). In low precipitation, dormancy percentage did not differ significantly between the two mucilage manipulations at any sand burial depth except 0 mm, where intact achenes had lower dormancy percentage than demucilaged ones (Figure 6A). In medium precipitation, dormancy percentages of intact achenes were significant lower than they were for demucilaged ones at 5 and 20 mm burial depths (Figure 6B). In high precipitation, dormancy percentages of intact achenes were significantly lower than they were for demucilaged ones at all burial depths (Figure 6C).

## Discussion

The present study addressed the role of seed mucilage in seedling emergence in different precipitation regimes and sand burial conditions that *A. sphaerocephala* encounters in the natural sand dune habitat. The low and unpredictable nature of precipitation in arid and semi-arid regions can limit successful seedling establishment, and water availability has been suggested to be one of the most important environmental factors for seedling emergence [30], [31]. Water-addition had positive effects on seedling emergence in four familial pairs of floodplain herbs [32]. In our study, cumulative seedling emergence in high water supply (July irrigation) was higher than that in medium or low water supply (June or May irrigation) for both types of achenes (Figure 2). Can mucilage enhance seedling emergence of *A. sphaerocephala* in different precipitation regime? We found that indeed, presence of mucilage increased seedling emergence (Figure 2) and this positive effect did not depend on water supply (Table 1). The enhancing effect of seed mucilage on seedling emergence may be due to its high water-retaining capacity under conditions of limited water supply [25]–[27], [33]. The next question in the present study is whether mucilage enhances seedling emergence under sand burial. We found significant interaction between mucilage manipulation and burial depth (Table 1), indicating that the benefit of mucilage depends on sand burial depth. In low irrigation, mucilage enhanced seedling emergence at all burial depths except 20 mm, where only a few seedlings emerged (Figure 2A, 2B). Furthermore, mucilage also promoted seedling emergence at 0 and 5 mm burial depth in medium irrigation and at 0 and 10 mm in high irrigation (Figure 2C, 2D, 2E, 2F). These results suggest that seed mucilage enhances seedling emergence in situations where water availability is low and at burial depths where there is a good supply of water.

Burial in sand causes changes in physical factors such as moisture, temperature, and aeration to which an organism is exposed [10]. Seedlings emergence from the soil depends not only on the energy within the seed but also burial depth [10], [34], [35]. We found that seedling emergence decreased in greater burial depth (Table 1; Figure 2) and ceased at a depth greater than 20 mm. This may result from the little increase in moisture with increasing depths (from 5 to 20 mm; Figure 1B), so negative effects of burial will very likely start to dominate when burial depth exceeded 5 mm. Maun showed that emergence of seeds of eight species was negatively correlated with seed burial depth [10]. A very similar relationship also has been reported for seeding emergence of *Leymus arenarius* [11]. Seedling emergence of *Leymus secalinus* in the same region as our study decreased as sand burial depth increased, and 1–2 cm was the optimal depth for seedling emergence [12], [30]. Huang and Gutterman studied seedling emergence in response to sand burial of a close relative of the test species of our study, *Artemisia monosperma*, and reported that the deeper the achenes in sand, the lower and slower their emergence [3].

Light is required for germination of *A. sphaerocephala* seeds [36]. However, spectrophotometric measurements have shown that less than 1% of the incident light penetrates 2.2 mm at any wavelength between 350 and 780 nm for ped sizes up to 1 mm [37] and that light penetration falls below 0.01% at a depth of 4 mm [38]. Therefore, absence of light, among other things, may be a cause for low emergence of buried achenes, but light could not be the main determinant of emergence of achenes buried in sand.

Additionally, it is well-known that large-seeded species or individuals of a species can emerge above the surface of the sand deposit at greater depths of burial [39]–[42]. In contrast, seedlings from small seeds that germinate deeply in soil may have high mortality percentages [2], [32], [43]–[45]. This might be the result of fewer resources stored in small seeds, which imposes a selection pressure that leads to very shallow maximum emergence depths for small seeds [42]. Because *A. sphaerocephala* has a 1000-seed mass of only 0.638±0.012 g [25] and needs light to geminate [36], the small seed mass may explain the low seedling emergence at deep sand burial depth.

Herbivory, drought and fungal attack are the main causes of seedling mortality [46], [47]. In our experiment, seedling mortality in the low irrigation treatment was significantly higher than that of medium and high irrigation (*P*<0.05; Figure 3), indicating that drought stress is the main cause of seedling mortality of *A. sphaerocephala* in this experiment. Seed mucilage had a positive effect on reducing seedling mortality, but this effect varied with sand burial depth (Figure 3, Table 1). High water-retaining capacity provided by the seed mucilage may reduce seedling mortality of *A. sphaerocephala*, but further study is needed to substantiate this. In our study, T_50_ of *A. sphaerocephala* was significantly earlier at 0 and 5 mm burial depth than it was at greater burial depths in high precipitation (*P*<0.05; Figure 4). Seedling emergence of *Cyperus capitatus* also was delayed as soil depth increased to 3 cm depth [48]. In general, intact achenes emerged earlier than those of demucilaged ones (Table 1; Figure 4), implying a role of seed mucilage in promoting the rate of seedling emergence in *A. sphaerocephala*.

Seed quiescence by sand burial may be related to low sand moisture content, low temperatures or poor aeration [30], [49]. In our study, quiescent achene percentages at 0 and 5 mm burial depths were significantly lower than they were at greater burial depths, which may be caused by the unsuitable condition for seedling emergence (i.e. low temperature, poor aeration). Seed mucilage had a significant effect on quiescence percentage, which varied with irrigation regime and sand burial depth (*P*<0.05; Table 1; Figure 5). Higher seed quiescence percentages were found in low and medium irrigations than in high irrigation, which may be due to more seeds that cannot emerge but remain quiescent in low and medium precipitation.

Freshly matured *A. sphaerocephala* seeds are not dormant [25]. Thus, seeds that were dormant at the end of the seedling emergence experiment were in secondary dormancy (induced dormancy). Induced dormancy percentage increased from 5 to 20 mm sand depths, and high proportions of achenes were in secondary dormancy at 20 mm burial depth in medium and high irrigations (*P*<0.05; Figure 6). Similarly, induced dormancy of *Psammochloa villosa* and *Leymus secalinus* caryopses increased with sand burial depth [12], [30]. A greater proportion of seeds of several sand dune species in Canada remained dormant at greater burial depth [15]. Similar results were reported for seed germination of three foredune plants, *Atriplex laciniata*, *Cakile maritima* and *Salsola kali* [14]. Pemadasa and Lovell speculated that the increase in percentage of seeds in induced dormancy with depth may be due to many factors, such as oxygen content, CO_2_ levels, aeration and sand water content [13]. This dormancy will benefit long-term survival of a species because an ungerminated seed maintained in the soil has the potential to produce a seedling at a later date when wind erosion reduces sand depth [10]. Furthermore, we found that seed mucilage had a significant effect on induced dormancy percentage, and fewer dormant achenes were observed for intact achenes than for demucilaged ones (Table 1; Figure 6), because more intact than demucilaged achenes emerged before dormancy percentage was tested.

Our recent reports have shown that seed mucilage of *A. sphaerocephala* can help maintain DNA integrity with the aid of desert dew or with small amounts of rain in the growing season [27], [28], aid seed germination in osmotically stressful and saline habitats [25] and promote early seedling growth in barren sand dunes [29]. In this study, we explored the role of mucilage in seedling emergence of this species under sand burial stress in three irrigation regimes that simulated the precipitation in seedling establishment and growing seasons in the natural habitat. Our results demonstrated that seed mucilage enhanced seedling emergence in *A. sphaerocephala* in precipitation regimes of seedling establishment and growth in the natural habitat and reduced seedling mortality at the shallow sand burial depth. Thus, seed mucilage plays an ecologically important role in successful seedling establishment of *A. sphaerocephala* by enhancing seedling emergence and reducing seedling mortality in stressful habitats of the arid sandy environment.

## Materials and Methods

### Ethics approval

The Ethics Review Boards of our institute (Academic Committee of Institute of Botany, the Chinese Academy of Sciences) and the Ordos Sandland Ecological Research Station of the Chinese Academy of Sciences have approved the study protocol. No specific permits were required for the described field studies. The location is not privately-owned or protected in any way, and the field studies did not involve endangered or protected species.

### Achene collection

In December 2010, freshly matured achenes of *A. sphaerocephala* were collected from dry unopened infructescences from natural populations near the Ordos Sandland Ecological Research Station of the Chinese Academy of Sciences (39°29′N, 110°11′E; 1296 m a.s.l.) on the Ordos Plateau in Inner Mongolia, north-central China (see a more complete description of this site in [25]). After they were brought to the laboratory, infructescences were shaken to detach the achenes, which were stored dry in a closed cotton bag at 5°C and 10% relative humidity until used in experiments.

### Mucilage removal

To remove the mucilage, intact achenes were submerged in water for 5–10 min and then rubbed gently and rapidly on filter paper several times, until no mucilage was released from them when they were imbibed with water [25], [27]. Hereafter, the achenes with mucilage removed are termed “demucilaged achenes”. Intact achenes used in the experiment were also treated with water in parallel with the demucilaged achenes.

### Sand burial and irrigation experiments

Raw sand was collected from sand dunes in the natural habitat of *A. sphaerocephala* and then sieved (2 mm mesh size) to remove the debris. The sand was dried in an oven for 24 h at 100°C to kill any viable seeds and soil microbes in it. Because soil temperature dramatically drops in a single inch depth in soils under high temperatures [50], [51], the sand was finely spread out (<2 cm) for drying and homogenized after the heating.

The seedling emergence experiment was conducted from 24 August to 23 September. Three irrigation regimes were applied in this study, namely 9.23 mm every 10 d, 11.01 mm every 8 d and 24.47 mm every 6 d, which simulated the precipitation amount and frequency in May, June and July, respectively, in the natural habitat (28.62 mm with frequency of 3.1 times in May; 40.72 mm with frequency of 3.7 times in June; and 110.13 mm with frequency of 4.5 times in July), based on the 10 year average of microenvironmental data obtained from the meteorological station established in the study area. The precipitation regimes in May, June and July were used because these three months are the most critical ones for seedling establishment and growth in the study area. Water was applied gently to avoid disturbing the seeds, especially those at the sand surface [52].

There were three irrigation regimes, six sowing depth treatments (0, 5, 10, 20, 40 and 60 mm) and four replicates per treatment for each mucilage manipulation. Thus, there were 144 pots in this experiment (2 mucilage manipulation×3 irrigation regimes×6 sowing depth×4 replicates). Replicates of 25 randomly selected seeds were sown in dry sand in drained cylindrical plastic pots (inner diameter, 80 mm; height, 80 mm). The pots were filled with prepared sand to the specific depth at which achenes were sown, and then the pots were filled with additional sand. The pots filled with sand were weighted to ensure the same amount of sand in each pot. The drainage outlet at the bottom of the pots was covered with cloth to prevent the loss of sand but not drainage of excess water. The pots with sand and seeds were placed in a random arrangement on a table in a non-heated greenhouse and the locations of pots were changed daily.

The pots were monitored every 24 h and the number of emerged and surviving seedlings counted. A seedling was considered to have emerged when its height exceeded 3 mm above the sand surface, and it was regarded to be dead when it fell over or had turned yellowish or brownish [8]. Seedling mortality was calculated on the basis of number of emerged seedlings. The time to reach 50% emergence (T_50_), which gave an estimate of emergence speed, was calculated according to the following formula of Coolbear et al. [53] modified by Farooq et al. [54]:
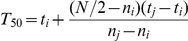
Where N is the final number of emergence and n_i_ and n_j_ the cumulative number of seeds germinated by adjacent counts at times t_i_ and t_j_ when n_i_<N/2<n_j_.

Temperature and relative humidity in the greenhouse were 17–29°C and 31–58%, respectively. To determine the fate of seeds that did not produce emerged seedlings in the 30-d experiment, seeds sown at different depths were dug out of the sand, and examined for evidence of germination. Ungerminated seeds were replanted at a depth of 0.5 cm in the same sterilized sand as above-mentioned with 3 mm water applied daily to ensure enough moisture for emergence, and seedling emergence was recorded daily for another 10 d. Seeds that produced emerged seedlings after replanting were considered to be quiescent [55]. Finally, the embryos of ungerminated seeds were cut into halves and soaked in 1% tetrazolium chloride (TTC) for 24 h at 25°C. Pink embryos were scored as viable, and viable seeds were considered to be dormant [55]. Quiescence and dormancy percentage were calculated as a percentage of the total number of seeds in the pot.

### Monitoring sand moisture

To monitor sand moisture in each irrigation regime, three extra pots without seeds were irrigated under the same conditions as those in each irrigation regime of the experiment. Each pot was weighed every day using an electronic balance (Mettler PM4600, Mettler Instrument AG, Germany). At the end of the experiment, sand at each of the different depth ranges in the pot was weighed to determine the moist sand weight, and the dry sand weight was determined after the sand was dried at 100°C for 24 h. Moisture content percentage (MC%) was calculated as:
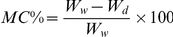
Where W_w_ is the weight of wet sand and W_d_ the weight of dry sand.

### Data analysis

No seedlings emerged from 40 or 60 mm burial depths; therefore, we collected data only for those buried at depths of 0 to 20 mm burial depths. Data in Figures are presented as arithmetic means ± SE. Soil moisture content was analyzed using two-way ANOVA, and irrigation regime and burial depth were treated as fixed factors. Seedling emergence data (final emergence percentage, T_50_, seedling mortality, quiescence and dormancy of achenes) were analyzed by three-way ANOVA, in which mucilage manipulation, irrigation regime and burial depth were treated as fixed factors. Post hoc comparisons were analyzed by Tukey's honestly significant difference. Differences between means were considered significant at the *P*<0.05 level. Proportions were arcsine transformed and soil moisture content and T_50_ square root transformed to ensure homogeneity of variance before subjecting them to statistical analysis. All statistical procedures were performed using SPSS Version 15.0 for Windows (SPSS Inc., Chicago, USA).
